# Analysis of potential factors influencing the willingness of Chinese freshmen to perform out-of-hospital CPR and use AED: a cross-sectional survey

**DOI:** 10.3389/fpubh.2026.1774036

**Published:** 2026-04-10

**Authors:** Yuanhua Hou, Ting Mao

**Affiliations:** Department of Internal Medicine, Lanzhou Jiaotong University Hospital, Lanzhou, China

**Keywords:** automated external defibrillator (AED), cross-sectional study, first-aid knowledge, out-of-hospital cardiopulmonary resuscitation (CPR), rescue willingness

## Abstract

**Background:**

Out-of-hospital cardiac arrest (OHCA) is a major public health issue in China. The average emergency response time of 16 min exceeds the 5-min “golden window.” Bystander CPR/AED use is crucial for survival. College freshmen are a key group for first-aid popularization, but their CPR/AED awareness, proficiency, willingness, and influencing factors are unclear. To investigate CPR/AED awareness, operational proficiency, rescue willingness, and influencing factors among college freshmen in Gansu, to inform optimized first-aid education.

**Methods:**

A cross-sectional study was conducted via convenience sampling on 1,443 first-year freshmen (August 30-September 1, 2023, Gansu). Eligible participants were officially enrolled freshmen with independent questionnaire completion ability; those with cognitive/language impairments were excluded. A structured questionnaire (referencing 2020 American Heart Association (AHA) Guidelines, reviewed by 3 associate senior experts) covered 5 sections. Distributed on-site during orientation, collected immediately (100% effective). Data were double-entered via EpiData 3.1, analyzed with SPSS 25.0.

**Results:**

Awareness was high (CPR: 87.87%; AED: 74.29%), but operational mastery was low (CPR: 34.37%; AED: 19.75%). Only 19.20% had prior training. Willingness to rescue was 59.60% (860/1443), with main barriers among unwilling participants being lack of skill (79.42%, 463/583) and perceived lack of first-aid qualifications (45.97%, 268/583). Multivariate analysis showed willingness to participate in future CPR training was positively associated with knowledge mastery (OR = 1.39, 95%CI: 1.09–1.76, *p* = 0.008), while prior training was a protective factor for knowledge mastery. For rescue willingness, protective factors included being female, mastering CPR/AED operation, understanding legal protections, and willingness for future training.

**Conclusion:**

Freshmen demonstrated high awareness but low proficiency and training coverage, with moderate rescue willingness. Recommendations include mandatory first-aid courses, increased AED deployment, and legal education. Study limitations include single-center design and self-reported data, which may lead to social desirability bias and limit the generalizability of the findings to universities in other regions of China.

## Introduction

Cardiac arrest refers to the sudden cessation of cardiac function and cardiac pump activity, leading to the cessation of breathing and circulation (1). Irreversible damage to cranial nerves occurs 5 min after cardiac arrest (2). As a global health issue and a major social burden, cardiac arrest is the primary cause of sudden death (3). Out-of-hospital cardiac arrest (OHCA) is defined as the loss of functional cardiac mechanical activity with no signs of circulation occurring outside of a hospital setting. In China, approximately 550,000 people suffer from OHCA annually (4).

Cardiopulmonary Resuscitation (CPR) is currently the main measure for rescuing cardiac arrest patients. Early identification of cardiac arrest and timely initiation of CPR can improve the survival rate of patients with cardiac arrest (5). However, studies have shown that most cardiac arrests occur outside hospitals (6). Emergency medical personnel cannot reach the scene immediately; one study found that the average time interval from call to the arrival of emergency personnel at the scene is 16 min (7). Therefore, early CPR performed by bystanders before the arrival of emergency personnel is of great importance. A study conducted in 8 large and medium-sized cities in China showed that the average rate of bystander-performed CPR was only 4.5% (4). Thus, improving bystanders’ knowledge and operational skills of CPR is crucial.

Cardiac arrest can occur in individuals of any age (8), including adults and students on campus (9). Colleges and universities are densely populated areas and high-risk locations for sudden cardiac death during physical activities. They also serve as important platforms for popularizing CPR-related knowledge. Popularizing CPR knowledge and skills among students is a key approach to improving the accessibility of bystander CPR (10), which not only enhances students’ self-rescue ability but also enables them to save others’ lives in emergencies. There is growing social support for incorporating CPR knowledge and skills into university curricula. At the 2000 International Conference on CPR Guidelines, experts reached a consensus that promoting basic CPR training in schools should be a fundamental education strategy. Many universities have begun to offer CPR health education courses, and some countries provide basic life support training (including AED use) to teaching staff (11). Baldi et al. (12) demonstrated that students require less time to learn Basic Life Support (BLS) than adults and achieve better results. The student population has strong learning and application abilities, generally high prosocial tendencies, and is among the most likely groups to provide bystander CPR in emergency settings (10–13). Despite the established importance of CPR training in college students, critical research gaps remain. First, most existing studies on CPR knowledge and willingness in Chinese college students focus on eastern and central China, with limited data on freshmen in northwestern provinces such as Gansu. Second, few studies have systematically characterized the gap between CPR/AED awareness and operational proficiency among Chinese freshmen, or identified the core barriers to bystander rescue in this population. Third, no studies to date have validated the mediating role of knowledge mastery in the association between CPR training and rescue willingness among Chinese college freshmen. Finally, existing questionnaires are primarily based on international guidelines, with limited adaptation to Chinese national CPR guidelines and the unique legal and cultural context of first aid in China. To address these gaps, this cross-sectional survey study was conducted to investigate the current status of CPR/AED awareness, skill mastery, and rescue willingness among college freshmen in Gansu Province, identify associated influencing factors, and provide evidence-based recommendations for optimizing college first-aid education systems in China.

## Methods

### Study subjects

A cross-sectional study design was adopted, with the subjects being first-year undergraduate freshmen officially enrolled in a university in Gansu Province, China, from August 30 to September 1, 2023. This group was selected because freshmen are in the early stage of adulthood, with strong learning and application abilities, and have not yet fully separated from the formal education environment. Their first-aid knowledge reserve and rescue willingness can reflect the popularization status of first-aid education in the previous 9-year compulsory education and high school stages. Meanwhile, this group has a relatively high probability of performing rescue in emergency scenarios, and the research results can provide direct references for optimizing the college first-aid training system and improving the OHCA rescue rate. A total of 4,218 freshmen were enrolled at the university’s main campus in 2023, covering all undergraduate majors (including engineering, science, management, literature, economics, and art, with no health science majors excluded). Participants were recruited via convenience sampling, with questionnaires distributed on-site during freshmen orientation. A total of 1,443 valid questionnaires were collected, with an overall response rate of 34.21% (1,443/4218). All returned questionnaires were complete for core items (no missing data among valid responses). Inclusion criteria: (1) First-year undergraduate freshmen officially enrolled at Lanzhou Jiaotong University from August 30 to September 1, 2023; (2) Aged 17–20 years; (3) Having basic reading and comprehension abilities to complete the questionnaire independently; (4) Voluntarily participating in the study and providing written informed consent. Exclusion criterion: Inability to complete the questionnaire independently due to cognitive impairment, language barriers, or other reasons. Participants voluntarily participated in this study with informed consent. The sample is representative of freshmen at science and engineering universities in Northwest China, with limited generalizability to other types of universities. This study involved human subjects and complied with the ethical requirements of the Declaration of Helsinki. The study protocol was approved by the Medical Ethics Committee of Lanzhou Jiaotong University Hospital (Approval No. [2023]012). All subjects signed informed consent forms. During the study, the privacy of the subjects was strictly protected; questionnaire data were only used for this study and anonymized to ensure data security.

### Study design type

This study was conducted at Lanzhou Jiaotong University, a provincial key comprehensive university in Gansu Province, northwest China. The university has 21 colleges, offering 68 undergraduate majors across engineering, science, management, literature, economics, law, and art disciplines, with a total of approximately 24,000 full-time undergraduate students. A cross-sectional study design was used. Through a one-time questionnaire survey, data on the demographic characteristics, CPR/AED-related knowledge and skills, rescue willingness, and training needs of the subjects were collected simultaneously, aiming to analyze the potential factors influencing freshmen’s performance of out-of-hospital CPR and AED use, and provide a basis for optimizing college first-aid training programs.

### Data collection tool

The questionnaire was a self-designed structured instrument informed by key recommendations from the 2020 American Heart Association (AHA) Guidelines for Cardiopulmonary Resuscitation and Emergency Cardiovascular Care (Adult BLS/ACLS). To enhance contextual relevance in China, item wording and scenarios were further adapted with reference to Chinese resuscitation-related consensus statements and the legal protection for voluntary emergency rescue under *China’s Civil Code* (Article 184, commonly referred to as the “Good Samaritan” provision). The questionnaire was designed and distributed via the online platform “Wenjuanxing,” a widely used professional questionnaire platform in China that supports mandatory item settings, logical skip and real-time data verification to ensure data quality. The questionnaire had good reliability and validity: the content validity index (CVI) was 0.92 (evaluated by 3 associate senior experts), and the Cronbach’s alpha coefficient was 0.85 (verified via pre-survey of 60 freshmen). The questionnaire consisted of 5 sections: 1. General information of respondents, including gender, age, and place of origin. 2. Awareness of CPR/AED (whether they had heard of CPR/AED, knew their specific operations) and previous participation in CPR/AED-related training. 3. First-aid legal knowledge and rescue willingness: Including two items on first-aid legal knowledge: (1) “Do you know the protective provisions of first-aid-related laws in the Civil Code of the People’s Republic of China (e.g., Article 184, the Good Samaritan provision)?” (5-point Likert scale: 1 = no knowledge at all, 5 = very familiar); (2) An open-ended question to describe the core content of the legal provisions. Rescue willingness was measured via the question “Are you willing to perform bystander rescue for a person experiencing out-of-hospital cardiac arrest?” (binary: yes/no), with a follow-up question on the reasons for unwillingness to rescue. 4. Specific CPR operations (9 questions: 1 sequencing question, 1 multiple-choice question, and 7 single-choice questions). 5. Willingness to participate in future CPR training and preferred training methods.

### Grouping criteria

To conduct univariate and multivariate analyses, two rounds of grouping were implemented based on the core outcome indicators of the study. Grouping by first-aid knowledge mastery: The first-aid knowledge questionnaire contained 9 questions (1 sequencing question, 1 multiple-choice question, and 7 single-choice questions). Participants who correctly answered ≥ 3 questions were classified into the well-mastered first-aid knowledge group (coded as 1), while those who correctly answered < 3 questions were assigned to the poorly-mastered first-aid knowledge group (coded as 0). This cut-off was determined based on pre-survey data and established methodologies in similar domestic college student CPR studies. Pre-survey analysis showed a statistically significant difference in knowledge levels between participants with ≥3 correct answers and those with <3 correct answers (*p* < 0.001). Grouping by rescue willingness: Based on the respondents’ answers to “Whether you are willing to rescue cardiac arrest patients”, students who answered “Yes” were included in the willing to rescue group (coded as 1), and those who answered “No” were placed in the unwilling to rescue group (coded as 0).

### Data collection process

Investigators received unified training to clarify the instructions for questionnaire completion, the standards for signing informed consent forms, and data recording requirements, so as to avoid survey bias. During the freshmen orientation, questionnaires were distributed on-site in centralized locations such as teaching buildings and dormitory areas. Investigators explained the research purpose, filling methods, and privacy protection measures on the spot to ensure that the subjects were fully informed. After the survey, questionnaires were collected immediately, and 2 investigators preliminarily verified the completeness and logic of the questionnaires, marked problematic questionnaires, and supplemented or excluded them in a timely manner to ensure the quality of original data.

### Statistical analysis

Valid questionnaire data were double-entered by two independent trained researchers using EpiData 3.1 software. After data entry, logical verification and data cleaning were performed to ensure data accuracy and consistency. No missing values were identified in the final dataset, as all core items were set as mandatory in the online questionnaire, and all collected questionnaires were fully completed. Therefore, no missing data processing was performed. Statistical analysis was performed using SPSS 25.0 software.

For quantitative data: the Shapiro–Wilk test was first used to determine whether the data conformed to a normal distribution. Normally distributed quantitative data were expressed as mean ± standard deviation, while skewed distributed quantitative data were described as median and interquartile range [M (P25, P75)]. Categorical data were presented as frequency (percentage, %).

For two groups of normally distributed quantitative data: Levene’s test was used to test for homogeneity of variance. If variance was homogeneous, the independent t-test was used; if variance was heterogeneous, the t’ test was used. Skewed distributed quantitative data or ordinal data were analyzed using the independent samples Mann–Whitney U test. The chi-square test or Fisher’s exact test was used to compare the composition ratios of qualitative data. Bonferroni correction was applied for multiple pairwise comparisons to reduce Type I error risk, with an adjusted *p*-value < 0.008 considered statistically significant for univariate analyses.

Binary Logistic regression analysis was used, with factors showing statistical significance in univariate analysis (*p* < 0.2) included in the multivariate Logistic regression. The forward stepwise (likelihood ratio) method was adopted for variable selection, and collinearity diagnostics were performed for all covariates (all variance inflation factor VIF < 2, no significant multicollinearity). Logistic regression was also used for mediating effect analysis. Mediation effect analysis was conducted to examine whether first-aid knowledge mastery mediated the association between prior first-aid training and rescue willingness. The causal steps approach was used, and the indirect effect was evaluated using the Sobel test and nonparametric bootstrapping (5,000 resamples) to obtain a 95% confidence interval. The mediation model was specified *a priori* based on a conceptual direction (prior training-knowledge mastery-rescue willingness), and we adjusted for measured potential confounders including age, gender, and place of origin. Given the cross-sectional design, mediation analysis was exploratory and does not establish temporal ordering or causality; results should be interpreted cautiously. All tests were two-tailed, and a *p*-value < 0.05 was considered statistically significant.

## Results

### Basic characteristics

From August 30 to September 1, 2023, a questionnaire survey was conducted among first-year undergraduate freshmen of a university in Gansu Province. A total of 1,443 questionnaires were distributed and recovered. The age of the subjects was (18.17 ± 0.78) years. In terms of gender distribution: 1,030 males (71.38%, 1,030/1,443) and 413 females (28.62%, 413/1,443). Regarding place of origin: 928 students (64.31%, 928/1,443) were from Northwest China, 199 (13.79%, 199/1,443) from East China, 124 (8.59%, 124/1,443) from Northeast China, and 96 each (6.65%, 96/1,443) from North China and Central South China (Table 1).

**Table 1:** Distribution of demographic characteristics, CPR/AED awareness and training status, rescue willingness, and training willingness.

**Variable**	**Total (n = 1,443)**
Age	18.17 ± 0.78
Gender	
Male	1,030 (71.38)
Female	413 (28.62)
Place of origin	
North China	96 (6.65)
Northeast China	124 (8.59)
East China	199 (13.79)
Central South China	96 (6.65)
Northwest China	928 (64.31)
Heard of CPR	1,268 (87.87)
Heard of AED	1,072 (74.29)
Know CPR operation	496 (34.37)
Know AED operation	285 (19.75)
Participated in first-aid training	277 (19.20)
Know protective provisions of first-aid laws	523 (36.24)
Willing to rescue	860 (59.60)
Unwilling to rescue	583 (40.40)
- Lack of operational skills	463 (79.42)
- Lack of confidence, dared not act	173 (29.67)
- Fear of causing secondary harm	208 (35.68)
- Unwilling to bear legal responsibility	38 (6.52)
- Fear of contracting diseases	10 (1.72)
- Believe no first-aid qualifications	268 (45.97)
Willing to participate in future CPR training	1,068 (74.01)
- Prefer popular science videos	782 (73.22)
- Prefer text introductions	270 (25.28)
- Prefer lectures	450 (42.13)
- Prefer simulated scenario training	502 (47.00)
- Prefer information retrieval	351 (32.87)

### Awareness of CPR/AED and training background

Among the 1,443 subjects:

- 87.87% (1,268/1,443) had heard of CPR, and 74.29% (1,072/1,443) had heard of AED. Only 34.37% (496/1,443) knew the specific operation of CPR, and 19.75% (285/1,443) knew the specific operation of AED.
- 277 students (19.20%, 277/1,443) had participated in first-aid-related training. Among them: 225 (81.23%, 225/277) through school-organized training, 22 (7.94%, 22/277) through self-directed video learning, 15 (5.42%, 15/277) through training by science and education public welfare organizations, 8 (2.89%, 8/277) through guidance from relatives or friends (doctors), and 7 (2.53%, 7/277) through other methods.
- 523 students (36.24%, 523/1,443) knew the protective provisions of first-aid laws, while 920 (63.76%, 920/1,443) did not (Table 1).

### Status of rescue willingness

Among the 1,443 subjects:

- 860 (59.60%, 860/1,443) stated that they were willing to rescue cardiac arrest patients, and 583 (40.40%, 583/1,443) were unwilling.

Among the 583 subjects unwilling to rescue:

- Ability-related reasons were the most common: 463 (79.42%, 463/583) reported “lack of operational skills”, 208 (35.68%, 208/583) “feared causing secondary harm”, and 173 (29.67%, 173/583) “lacked confidence and dared not act”.
- Concern-related reasons: 38 (6.52%, 38/583) “unwilling to bear legal responsibility” and 10 (1.72%, 10/583) “feared contracting diseases”.
- Qualification cognition-related reasons: 268 (45.97%, 268/583) “believed they did not have first-aid qualifications” (Table 1).

### Mastery of first-aid knowledge

The first-aid knowledge questionnaire contained 9 questions (1 sequencing question, 1 multiple-choice question, and 7 single-choice questions). The average number of correctly answered questions by the subjects was (2.62 ± 1.27).

The correct rate for chest compression-to-ventilation ratio was the highest (27.03%, 390/1,443), whereas the correct rate for correctness of step sequencing was the lowest (0.83%, 12/1,443). Among the single-item CPR knowledge questions, the correct rate for CPR procedure was the lowest (5.89%, 85/1,443). Taking “correctly answering ≥ 3 questions” as “well-mastered” and “< 3 correct answers” as “poorly-mastered,” among the 1,443 subjects, 767 were classified as “well-mastered” and 676 (1,443–767 = 676) as “poorly-mastered” (Table 1).

### Univariate analysis of first-aid knowledge mastery

Univariate analysis results showed that age, gender, and place of origin had no statistically significant effects on the level of first-aid knowledge mastery (all *p* > 0.05). Factors with statistically significant effects (all *p* < 0.05) included: having heard of AED, knowing CPR operation, knowing AED operation, knowing protective provisions of first-aid laws, having participated in first-aid training, and willingness to participate in future CPR training (Table 2).

**Table 2:** Univariate analysis of first-aid knowledge mastery level.

Demographic variable	Poorly-mastered (*n* = 676)	Well-mastered (*n* = 767)	Statistic	*P*-value
Age	18.19 ± 0.81	18.16 ± 0.74	0.76	0.445
Gender			0.16	0.685
- Male	486 (71.89)	544 (70.93)		
- Female	190 (28.11)	223 (29.07)		
Place of origin			5.72	0.221
- North China	41 (6.07)	55 (7.17)		
- Northeast China	50 (7.40)	74 (9.65)		
- East China	93 (13.76)	106 (13.82)		
- Central South China	39 (5.77)	57 (7.43)		
- Northwest China	453 (67.01)	475 (61.93)		
Heard of CPR	582 (86.09)	686 (89.44)	3.77	0.052
Heard of AED	480 (71.01)	592 (77.18)	7.18	0.007
Know CPR operation	201 (29.73)	295 (38.46)	12.13	<0.001
Know AED operation	111 (16.42)	174 (22.69)	8.90	0.003
Know protective provisions of first-aid laws	225 (33.28)	298 (38.85)	4.82	0.028
Participated in first-aid training	580 (85.80)	586 (76.40)	20.46	<0.001
Previous training methods			–	0.809
- School-organized training	77 (80.21)	148 (81.77)		
- Self-directed video learning	9 (9.38)	13 (7.18)		
- Science and education public welfare organizations	4 (4.17)	8 (4.42)		
- Guidance from relatives/friends (Doctors)	0 (0.00)	3 (1.66)		
- Others	6 (6.25)	9 (4.97)		
Willing to participate in future CPR training	473 (69.97)	595 (77.57)	10.80	0.001

### Multivariate unconditional logistic regression analysis of first-aid knowledge mastery

Taking “whether first-aid knowledge is mastered” as the dependent variable (well-mastered = 1, poorly-mastered = 0), and including variables with statistical significance in univariate analysis (having heard of AED, willingness to participate in CPR-related training, having participated in first-aid training) as independent variables, multivariate unconditional Logistic regression analysis was conducted. Results showed that willingness to participate in CPR-related training was positively associated with first-aid knowledge mastery (OR = 1.39, 95% CI: 1.09–1.76, *p* = 0.008). Having participated in first-aid training was a protective factor for first-aid knowledge mastery (OR = 0.57, 95% CI: 0.43–0.75, *p* < 0.001). Having heard of AED had no statistically significant effect on first-aid knowledge mastery (OR = 1.27, 95% CI: 1.00–1.61, *p* = 0.052) (Table 3).

**Table 3:** Multivariate unconditional logistic regression analysis of first-aid knowledge mastery level.

Variable	*β*	S.E	*Z*	*P*	OR (95% CI)
Heard of AED	0.24	0.12	1.94	0.052	1.27 (1.00–1.61)
Willingness to participate in CPR-related training	0.33	0.12	2.67	0.008	1.39 (1.09–1.76)
Having participated in first-aid training	−0.56	0.14	−3.97	<0.001	0.57 (0.43–0.75)

### Univariate analysis of rescue willingness

Univariate analysis results showed that age, gender, and place of origin had no statistically significant effects on rescue willingness (all *p* > 0.05). Factors with statistically significant effects (*p* < 0.05) included: having heard of CPR, having heard of AED, knowing CPR operation, knowing AED operation, knowing protective provisions of first-aid laws, having participated in first-aid training, and willingness to participate in future CPR training (Table 4).

**Table 4:** Univariate analysis of rescue willingness.

Variable	Willing to rescue (*n* = 860)	Unwilling to rescue (*n* = 583)	Statistic	*P*
Age	18.19 ± 0.76	18.15 ± 0.79	0.99	0.321
Gender			3.23	0.072
- Male	629 (73.14)	401 (68.78)		
- Female	231 (26.86)	182 (31.22)		
Place of origin			3.22	0.522
- North China	56 (6.51)	40 (6.86)		
- Northeast China	71 (8.26)	53 (9.09)		
- East China	123 (14.30)	76 (13.04)		
- Central South China	50 (5.81)	46 (7.89)		
- Northwest China	560 (65.12)	368 (63.12)		
Heard of CPR	774 (90.00)	494 (84.73)	9.04	0.003
Heard of AED	669 (77.79)	403 (69.13)	13.66	<0.001
Know CPR operation	388 (45.12)	108 (18.52)	108.91	<0.001
Know AED operation	243 (28.26)	42 (7.20)	97.15	<0.001
Know protective provisions of first-aid laws	409 (47.56)	114 (19.55)	117.92	<0.001
Participated in first-aid training	642 (74.65)	524 (89.88)	51.95	<0.001
Training methods				0.002
- School-organized training	181 (83.03)	44 (74.58)		
- Self-directed video learning	21 (9.63)	1 (1.69)		
- Science and education public welfare organizations	7 (3.21)	5 (8.47)		
- Guidance from relatives/friends (Doctors)	2 (0.92)	1 (1.69)		
- Others	7 (3.21)	8 (13.56)		
Willing to participate in future CPR training	682 (79.30)	386 (66.21)	30.97	< 0.001
Number of correct answers	2.66 ± 1.28	2.58 ± 1.25	1.16	0.245
Correctness of step sequencing	10 (1.16)	2 (0.34)	1.92	0.165
CPR components	497 (57.79)	345 (59.18)	0.27	0.600
Chest compression site	481 (55.93)	309 (53.00)	1.20	0.273
Chest compression rate	291 (33.84)	198 (33.96)	0.00	0.961
Chest compression depth	275 (31.98)	167 (28.64)	1.82	0.178
CPR procedure	57 (6.63)	28 (4.80)	2.09	0.148
Airway opening methods	329 (38.26)	204 (34.99)	1.59	0.207
Chest compression-to-ventilation ratio	261 (30.35)	129 (22.13)	11.91	< 0.001

① Measurement scale: Age was a continuous variable; gender, place of origin, CPR/AED awareness, operational mastery, legal knowledge, training history, and individual question correctness were binary categorical variables; number of correct answers was a continuous variable. ② Statistical methods: Independent samples *t*-test was used for normally distributed continuous variables; Mann–Whitney *U* test was used for skewed continuous variables and ordinal data; χ^2^ test was used for binary categorical variables. ③ Bonferroni correction was applied for multiple comparisons.

### Multivariate unconditional logistic regression analysis of rescue willingness

Taking “whether willing to rescue” as the dependent variable (willing = 1, unwilling = 0), and including variables with statistical significance in univariate analysis as independent variables, multivariate unconditional Logistic regression analysis was conducted. Protective factors for rescue willingness included being female (*p* = 0.006), knowing specific CPR operation (*p* < 0.001), knowing specific AED operation (*p* = 0.003), knowing protective provisions of first-aid laws (*p* < 0.001), and willingness to participate in CPR-related training (*p* < 0.001). Having participated in first-aid training (*p* = 0.082) and knowing chest compression-to-ventilation ratio (*p* = 0.094) had no statistically significant effects on rescue willingness (Table 5).

**Table 5:** Multivariate unconditional logistic regression analysis of rescue willingness.

Variable	*β*	S.E	*Z*	*P*	OR (95% CI)
Being female	−0.36	0.13	−2.77	0.006	0.70 (0.54–0.90)
Knowing specific CPR operation	−0.66	0.15	−4.31	<0.001	0.52 (0.38–0.70)
Knowing specific AED operation	−0.63	0.21	−2.92	0.003	0.53 (0.35–0.81)
Knowing protective provisions of first-aid laws	−0.84	0.14	−5.89	<0.001	0.43 (0.33–0.57)
Willingness to participate in CPR-related training	−0.65	0.13	−4.97	<0.001	0.52 (0.40–0.67)
Having participated in first-aid training	0.32	0.18	1.74	0.082	1.37 (0.96–1.96)
Knowing chest compression-to-ventilation ratio	0.23	0.14	1.68	0.094	1.25 (0.96–1.63)

### Mediating effect analysis of the “training-knowledge mastery-rescue willingness” pathway

This analysis aimed to verify whether “having participated in first-aid training (explanatory variable, X)” affects “whether willing to rescue (outcome variable, Y; coded as 1 = willing, 0 = unwilling)” through “well-mastered first-aid knowledge (mediating variable, M; i.e., ‘more correct answers’)”.

Results showed that students who had participated in first-aid training were more likely to have well-mastered first-aid knowledge than those who had not, and this difference was not accidental. Students who had participated in first-aid training had a significant direct effect on “whether willing to rescue”. However, whether students mastered first-aid knowledge well or poorly did not change their “willingness to rescue”; the level of knowledge mastery was not a factor influencing rescue willingness.

These results indicated that the effect of prior first-aid training on rescue willingness was not transmitted through the mediating variable of first-aid knowledge mastery. The Sobel test confirmed no significant indirect effect (*Z* = 0.01, *p* = 0.991), and bootstrapping analysis (5,000 samples) showed that the 95% CI for the indirect effect (−0.21 to 0.22) included 0, further confirming the absence of a significant mediating effect. These findings indicate that we found no evidence that prior first-aid training improves students’ rescue willingness by enhancing their first-aid knowledge mastery in this sample. First-aid knowledge mastery showed no significant independent association with rescue willingness, suggesting that students’ willingness to perform bystander rescue may be driven primarily by non-knowledge factors such as operational confidence, legal cognition, and perceived professional qualifications, rather than theoretical knowledge level alone. Potential suppressor effects and unmeasured confounding factors cannot be excluded (Table 6).

**Table 6:** Mediating effect analysis of the “first-aid training-knowledge mastery-rescue willingness” pathway.

Path	Relationship	*β*	SE	Lower 95% CI	Upper 95% CI	*P*	*β* (95% CI)
Training → Knowledge mastery	Explanatory → Mediating	0.62	0.14	0.35	0.90	<0.001	0.62 (0.35–0.90)
Training → Rescue willingness	Explanatory → Outcome	−1.10	0.16	−1.42	−0.79	<0.001	−1.10 (−1.42–-0.79)
Knowledge mastery → Rescue willingness	Mediating → Outcome	0.00	0.11	−0.21	0.22	0.991	0.00 (−0.21–0.22)

## Discussion

Out-of-Hospital Cardiac Arrest (OHCA) is a major public health challenge in China. A study by Shao et al. (7) showed that 74.5% of cardiac arrests occur at home and 15.5% in public places, further confirming that OHCA is the main scenario for cardiac arrest. The average time for emergency medical personnel to arrive at the scene is 16 min, far exceeding the 5-min “golden rescue window” after cardiac arrest. Bystander-performed CPR has therefore become a key link in improving the survival rate of OHCA patients.

College students, as a young group, have strong learning abilities and high potential willingness to rescue (12), making them a core target for popularizing CPR and Automated External Defibrillator (AED) knowledge and skills. This study conducted a cross-sectional survey of the 2023 cohort of freshmen at a university in Gansu Province, analyzing their current status of CPR/AED awareness, skill mastery, and rescue willingness, and comparing the results with domestic and international studies. The aim was to provide a basis for optimizing the college first-aid training system, and the results are discussed below in conjunction with existing issues.

This study showed that 87.87% of freshmen had heard of CPR, a significantly higher awareness rate than the 28% reported in a 2010 study by Chen et al. (14) on Chinese students. This reflects the effectiveness of social-level first-aid popularization (e.g., media publicity, health courses in compulsory education) over the past decade. However, there was a clear disconnect between awareness and practical ability: only 34.37% of freshmen knew the specific operation of CPR, and 19.20% had participated in CPR-related training. Although this training rate was higher than the 14.6% reported in a 2016 study by Huang et al. (15) and 3% in the 2010 study by Chen et al. (14), it remained lower than the 34.8% in a 2020 study by Yi Jiang et al. (16), 33.6% in a 2021 study by Mao et al. (17) in Chongqing, and 26% in a 2017 study by Chen et al. (18). This indicates a significant gap in CPR training coverage among college freshmen.

The gap in AED-related cognition was even more prominent: 74.29% of freshmen had heard of AED, far higher than the 31.0% reported in an international study by Kwiecień-Jaguś et al. (19) on nursing students. However, only 19.75% knew the specific operation of AED, and 2.3% had participated in AED training, significantly lower than the results of a study by Wang et al. (20) on e-learners of first aid. This characteristic of “better CPR cognition than AED cognition” is consistent with a community study in Nantong, China (21). The presumed reasons are: CPR popularization started earlier and does not require equipment support, while the deployment rate of AEDs in public places (e.g., insufficient AED coverage in colleges) is low. Additionally, AED training is mostly bundled with CPR training but not separately emphasized, leading to a breakdown in the “cognition-operation” chain for AED among students.

Overall, the core reason for “high awareness, low operational proficiency, and low training coverage” lies in the fragmentation of previous first-aid education: most popularization efforts remain at the theoretical level of “knowing about CPR/AED”, lacking standardized practical training. As a result, students only form “cognitive memory” but cannot translate it into practical operational ability. Furthermore, colleges and universities have not incorporated first-aid training into compulsory courses, relying only on short-term voluntary lectures, which further reduces the coverage and effectiveness of training.

In this study, 59.6% of freshmen were willing to rescue OHCA patients. This proportion is close to the 59.7% reported in a 2016 study by Huang et al. (15) in China and 68.0% in a study by De Smedt et al. (22) in Belgium, higher than the 34.1% in a 2021 study by Mao et al. (17) in Chongqing, 44.4% in a study by Lu et al. (23) in Tianjin, and 37.4% in a study by Karuthan et al. (24) in Malaysia, but lower than the 72.3% in a study by Wanke et al. (25) in Germany and 90.10% in a study by Wang et al. (20) in Wuhan, China. This reflects that the rescue willingness of Chinese college freshmen is at a moderate level, with room for improvement.

From the perspective of reasons for unwillingness to rescue, skill deficiency was the primary barrier: 79.42% of freshmen cited “lack of operational skills”, consistent with the findings of studies by Karuthan et al. (24) in Malaysia, Al-Turki et al. (26) in Saudi Arabia, and Shams et al. (27) in Lebanon. This confirms that “insufficient ability” is a common issue restricting bystander rescue. The second most common reason was “believing they do not have first-aid qualifications” (45.97%), which is related to the traditional Chinese perception that “professional tasks require professional personnel” — most students fail to recognize the “timeliness value” of bystander first aid and overlook the characteristic that “early intervention is better than delayed professional intervention” in OHCA rescue. In addition, “fear of causing secondary harm” (35.68%) and “lack of confidence” (29.67%) also accounted for relatively high proportions, similar to the results of studies by Wang et al. (22) (59.30% feared harming patients) and Mao et al. (19) (66.6% lacked confidence). Even medical students have similar concerns (28), suggesting that “operational confidence building” should be included as a training focus.

Notably, “unwillingness to bear legal responsibility” accounted for only 6.7% in this study, far lower than the 20.7% in the study by Yi Jiang et al. (16), 26.74% in the study by Wang et al. (20), and 37.4% in the study by Mao et al. (17). This is presumably due to the widespread social publicity of China’s “Good Samaritan Law” in recent years, which has implicitly reduced students’ overall legal concerns about rescue. However, 63.76% of freshmen still did not know the specific protective provisions of first-aid laws, indicating a critical gap in systematic first-aid legal education among college freshmen. Social-level publicity cannot replace targeted, systematic legal education within university first-aid training programs, which is essential to further reduce legal barriers to bystander rescue. “Fear of contracting diseases” accounted for 2.4%, which, although lower than in some studies (19, 25), still requires the inclusion of “protective measures” (e.g., use of simple breathing barriers) in training to further eliminate this concern.

Based on the Logistic regression model, this study found that gender is one of the independent influencing factors of rescue willingness, which is inconsistent with the study by Karuthan et al. (24) in Malaysia. However, some studies have reported similar results: a study by Mao et al. (17) in Chongqing showed that females are more willing to provide simple help, while a study by Jtga et al. (29) in Austria found that males are more willing to perform CPR. This discrepancy is presumably related to the regional culture of the sample and training experience — the sample in this study was mainly from a university in Gansu, and there was no significant difference in previous first-aid popularization received by male and female freshmen, so no gender differentiation was observed.

In this study, the average number of correctly answered questions in the CPR knowledge questionnaire among freshmen was only (2.62 ± 1.27). Even the question with the highest correct rate — “chest compression-to-ventilation ratio” (27.03%) — was lower than the 80.9% reported in a study by Kwiecień-Jaguś et al. (19) in Poland, and only higher than the 16.9% in a study by Mansour et al. (30) in Saudi Arabia. The correct rate of operation sequencing was extremely low, with only 0.83% of freshmen able to correctly sequence CPR operation steps, far lower than the 22.6% reported in a study by Kwiecień-Jaguś et al. (19) on nursing students. This reflects a serious lack of understanding of the “logical flow of CPR procedures” among students. Mastery of basic knowledge points was uneven: 54.75% knew the correct compression site, 34.03% knew the correct compression rate, 30.63% knew the compression depth, and 27.1% knew the compression-to-ventilation ratio.

The reason for this “fragmented mastery” is that previous first-aid education mostly focused on “knowledge point indoctrination” (e.g., only informing that “the compression site is between the two nipples”) rather than helping students establish a complete logic of “assessment-call-operation” through “procedural practical training”. As a result, students can memorize individual knowledge points but cannot integrate them into practical operational ability. In addition, the “forgetting effect” of CPR knowledge cannot be ignored — most students only encountered first-aid knowledge in middle school and did not reinforce it through retraining, further reducing knowledge retention.

In this study, 74.01% of freshmen were willing to participate in CPR training, a proportion close to the 77% reported in the 2010 study by Chen et al. (14), higher than the 45.5% in the 2021 study by Mao et al. (17) in Chongqing, but lower than the 82.5% in the study by Mansour et al. (30) in Saudi Arabia, 88.3% in the study by Huang et al. (15), and 92.6% in the study by Lu et al. (25) in Tianjin. This suggests that college freshmen have a strong demand for training, but training methods need to be optimized to enhance participation enthusiasm.

In terms of preferred training methods, freshmen prioritized “popular science videos” and “simulated scenario training”, while traditional methods such as “attending lectures” and “reading text introductions” were less attractive. This trend is consistent with the evolution of first-aid knowledge acquisition channels: a 2010 study by Chen et al. (14) showed that students mainly obtained first-aid knowledge through TV (39%) and books (29%); a 2016 study by Huang et al. (15) still found TV and books as the main channels; however, a 2021 study by Qian et al. (23) in Nantong showed that 61.98% of residents obtained knowledge through online platforms. The popularity of electronic devices has made students more inclined to “intuitive and interactive” learning, and traditional “cramming-style” lectures can no longer meet their needs.

Based on this, college first-aid training should establish an “online + offline” integrated model: push CPR/AED popular science videos through school official accounts and short-video platforms (to match students’ preferences) online; collaborate with affiliated hospitals and the Red Cross to conduct “simulated scenario training” offline (e.g., simulating OHCA scenes to practice the full process of “assessment-call-compression-defibrillation”); and establish a “training-assessment-retraining” closed loop (retraining once per semester) to avoid knowledge forgetting. Additionally, efforts should be made to strengthen AED popularization and training: deploy AEDs in public places on campus (e.g., teaching buildings, gymnasiums) and emphasize AED operation independently in training (e.g., identifying electrode pad positions and operation procedures) to achieve “CPR + AED” coordinated training.

The results of the mediating effect analysis showed that for college freshmen, “willingness to rescue” depends not only on “ability to rescue” (knowledge/skills) but also on “courage to rescue” (confidence, legal cognition). Even if first-aid knowledge is mastered, students may still avoid rescuing if they have psychological barriers such as “fear of causing secondary harm” or “believing they lack qualifications”. For childcare staff, although Xia Lin’s study (31) did not conduct mediating effect analysis, it found that “having learned first-aid skills” was the strongest protective factor for skill mastery (OR = 12.486, *p* < 0.05), indicating the direct role of training in improving skills. However, whether skills can be converted into rescue behavior also depends on non-knowledge factors such as “confidence” and “scenario adaptability”.

This study has several limitations that should be acknowledged. First, this was a single-center study conducted at a science and engineering university in northwestern China, with the majority of participants from Northwest China. The findings may not be generalizable to medical universities, liberal arts universities, or institutions in other regions of China. Second, data were collected via self-reported questionnaires, which may introduce recall bias and social desirability bias, potentially leading to overestimation of self-reported rescue willingness and perceived operational familiarity. Third, the cross-sectional design limits inference on temporal ordering and prevents causal conclusions regarding the relationships among prior training, knowledge mastery, and rescue willingness. Fourth, CPR and AED operational competence was assessed using theoretical knowledge items and a single binary self-assessment question (yes/no) on whether participants knew the specific operations, rather than standardized performance-based evaluations. Therefore, our findings primarily reflect perceived competence and knowledge, and may not fully capture actual hands-on CPR/AED skills. Future studies should incorporate objective assessments, such as scenario-based simulations or manikin-based skill testing with standardized checklists. Finally, multiple univariate comparisons were performed, and while Bonferroni correction was applied, the risk of Type I error cannot be completely eliminated.

## Conclusion

This study only included freshmen from a single university in Gansu Province, with most students from Northwest China. As a result, the findings cannot be generalized to all universities in China. Future studies should expand the sample size to include universities in different regions and of different types. The cross-sectional design limits the ability to determine the causal relationship between “training-knowledge-willingness,” and cohort studies are needed for further verification. Additionally, there is potential bias from self-reported data, as skill mastery was assessed through self-completed questionnaires, which may lead to “overestimation of ability”. Future studies should combine practical operation assessments to improve data accuracy.

Incorporate first-aid training into compulsory college courses, using the 2020 American Heart Association Guidelines for Cardiopulmonary Resuscitation as the standard, covering both CPR/AED theory and practice, to ensure 100% participation of freshmen and address insufficient training coverage. Optimize training content and forms: push “popular science videos + legal cases” (e.g., practical cases of the “Good Samaritan Law”) online; conduct “simulated scenario training” offline (e.g., simulating OHCA scenes); and include “protective measure” teaching to eliminate skill, legal, and health concerns. Strengthen AED popularization and training: deploy AEDs in public places on campus (teaching buildings, gymnasiums) and emphasize AED operation independently in training (e.g., identifying electrode pad positions and operation procedures) to achieve “CPR + AED” coordinated training. Establish a retraining and assessment mechanism: conduct first-aid knowledge retraining once per semester, and require students to pass a “theoretical + practical” assessment to complete the course, ensuring training quality.

## Data Availability

The original contributions presented in the study are included in the article/supplementary material, further inquiries can be directed to the corresponding author.
